# Combining Ascending Aortic Diameter and Length and Their Growth Rates Improves the Prediction of Type A Aortic Dissection

**DOI:** 10.1093/ejcts/ezag110

**Published:** 2026-02-27

**Authors:** Iida-Mari Kotanen, Tuomas Selander, Christian Olsson, Anders Franco-Cereceda, David Marlevi, Petri Saari, Tarmo Korpela, Saara Sillanmäki, Marja Hedman

**Affiliations:** Department of Cardiothoracic Surgery, Heart Centre, Kuopio University Hospital (KUH), Kuopio 70210, Finland; Science Service Centre, KUH, Kuopio 70210, Finland; Department of Cardiothoracic Surgery, Karolinska University Hospital, Stockholm 171 76, Sweden; Department of Cardiothoracic Surgery, Karolinska University Hospital, Stockholm 171 76, Sweden; Department of Molecular Medicine and Surgery, Karolinska Institute, Stockholm 171 65, Sweden; Clinical Imaging Centre, KUH, Kuopio 70210, Finland; Department of Cardiothoracic Surgery, Heart Centre, Kuopio University Hospital (KUH), Kuopio 70210, Finland; Clinical Imaging Centre, KUH, Kuopio 70210, Finland; Institute of Clinical Medicine, University of Eastern Finland, Kuopio 70210, Finland; Department of Cardiothoracic Surgery, Heart Centre, Kuopio University Hospital (KUH), Kuopio 70210, Finland; Institute of Clinical Medicine, University of Eastern Finland, Kuopio 70210, Finland

**Keywords:** ascending aortic diameter, ascending aortic length, ascending thoracic aortic aneurysm, acute type A aortic dissection, growth rate, risk evaluation

## Abstract

**Objectives:**

The majority of acute type A aortic dissections (ATAADs) occur at ascending aortic dimensions and growth rates below current preventive surgery thresholds. This study aimed to investigate the risk of ATAAD based on the ascending aortic diameter, length, and their growth rates.

**Methods:**

The ascending aortic diameters and lengths were measured using repeated pre-dissection computed tomography and magnetic resonance imaging acquired during follow-up to evaluate their growth patterns and rates prior to the ATAAD onset. Measurements and growth rates were used to develop the risk function for the ATAAD risk assessment.

**Results:**

Ascending aortic aneurysm patients (*n* = 116) were divided into ATAAD (*n* = 30) and non-ATAAD groups (*n* = 86). Almost half of the ATAAD patients (46.7%) did not exceed the current preventive surgery thresholds based on the dimensions. A diameter growth rate of 3 mm/year was extremely rare (2.6%). The risk function was introduced in this study, and it could be used to predict the 5-year risk of ATAAD with excellent confidence (area under the curve [AUC] value of 0.83 (95% CI [0.54-0.98])).

**Conclusions:**

The combination of ascending aortic diameter and length and their growth rates offers a valuable tool for assessing the risk of an ATAAD. Almost half of the ATAAD patients fall below the established thresholds when assessed using only ascending aortic dimensions. These findings suggest that preventive surgery thresholds may benefit from including all four parameters: ascending aortic diameter and length and their growth rates.

**Clinical registration number:**

DilAo-Trial ClinicalTrials.gov ID 5063566

## Graphical abstract

**Figure:**
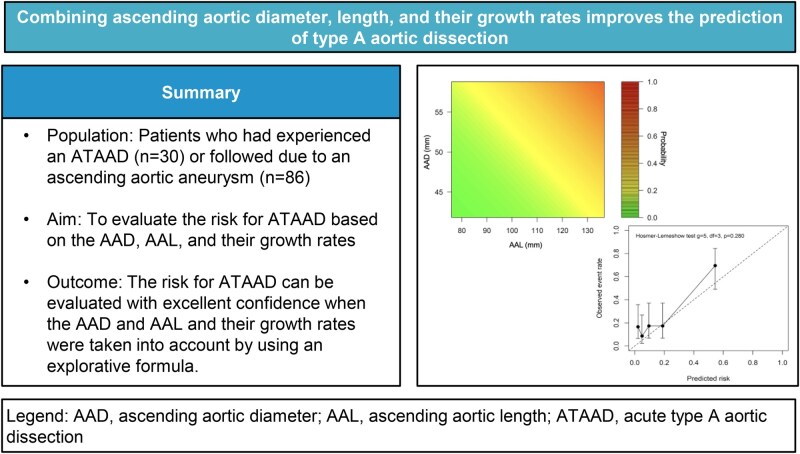

## Introduction

Ascending thoracic aortic aneurysm (ATAA) has been determined to be one of the main risk factors for acute type A aortic dissection (ATAAD). The annual risk of ATAAD exceeds 1% when the ascending aortic diameter (AAD) reaches 50-54 mm.^1^ According to current guidelines, the threshold for preventive surgery is an AAD of 50-55 mm, with consideration of the AAD growth rate and ascending aortic length (AAL).^2^^,^^3^ For the AAD growth rate, the threshold is over 3 mm/year,^2^^,^^3^ although high AAD growth rates are rare.^1^^,^^4^^,^^5^ Patients with ATAAD have longer AALs than those with ATAA without dissection.^6^^,^^7^ Moreover, the risk of ATAAD increases together with the AAL, with the critical point of >110 mm.^2^^,^^3^

Most ATAADs occur at AADs that do not meet the criteria for preventive surgery,^8–10^ and very little is known about the AAD growth rate before ATAAD occurs. Also, a pre-dissection AAL shorter than 110 mm is common among ATAAD patients.^11^ Therefore, further studies are needed to identify more accurate predictors of ATAAD.

This study aimed to investigate AAD and AAL in patients with ATAAD on the day of ATAAD onset using a linear model based on repeated computed tomography (CT) and magnetic resonance imaging (MRI). It also aimed to investigate AAD and AAL growth rates prior to the ATAAD onset, in order to evaluate the individual risk for an ATAAD. Previous studies have used pre-dissection images to explore pre-dissection aortic dimensions and the risk of ATAAD.^9^^,^^12^^,^^13^ In this study, only pre-dissection imaging was used for the ATAAD risk evaluation.

## Methods

### Ethical statement

This study was approved by the Ethical Committee of Wellbeing County of North Savo (200/2017) and by the Stockholm Regional Ethical Committee (2012/1633-31/4). The study followed the Declaration of Helsinki. All patients in the non-ATAAD group had given their informed written consent (clinicaltrials.gov ID: 5063566), and all registry data regarding the ATAAD group had been collected under institutional permission.

### Patient population

This was a collaborative study between Karolinska University Hospital (KaUH), Stockholm, Sweden, and Kuopio University Hospital (KUH), Kuopio, Finland. The study included two patient groups: ATAAD and non-ATAAD. ATAAD patients had been operated on at KaUH between January 2006 and December 2022 or at KUH between February 2009 and December 2021. Non-ATAAD patients were followed due to an ATAA at KUH between July 2003 and October 2024. Exclusion criteria were as follows: (1) fewer than two pre-dissection CTs and/or MRIs during the surveillance period, (2) surveillance period shorter than one year, (3) an iatrogenic ATAAD, and (4) non-ATAAD patients with normal AAD (< 40 mm) in our measurements. A total of 116 patients were included (ATAAD: *n* = 30 and non-ATAAD: *n* = 86).

Baseline characteristics were obtained from medical records, including data on comorbidities, height, weight, smoking history, family history of aortic diseases, genetic predisposition to ATAA, and aortic valve morphology. *Hypertension* was defined as the use of antihypertensive medication, and *dyslipidaemia* as the use of lipid-lowering therapy. Current or former smokers were classified as *smokers*. The morphology of the aortic valve was evaluated using echocardiography. *Other aortic diseases* included an abdominal aortic aneurysm and a chronic type B dissection diagnosed before an ATAAD onset.

### Image analysis

As there is excellent agreement in thoracic aortic measurements between CT and MRI,^14^ all CT and MRI scans covering the chest area obtained prior to ATAAD onset were included, regardless of the imaging indication. The maximum AAD and AAL values were measured from all images.

A picture archiving and communication system (PACS; Sectra vs 24.2) was used to measure AADs. Multiplanar reconstruction of the image stacks was used to allow a three-dimensional presentation of the structures. AADs were measured by a single author (I-M.K.) using the inner-to-inner edge method.^2^ Measurements were repeated at the level of the largest AAD and taken perpendicularly to the direction of the blood flow. AADs were measured in three directions at the level of the sinus of Valsalva and in two directions at other parts of the ascending aorta. The technique of measuring is explained in more detail in a previous study.^15^

The AAL measurements were performed with syngo.via (Siemens Healthineers, Erlangen, Germany). The AAL was measured as the distance between the aortic annulus and the origin of the brachiocephalic trunk in the centre line of the aorta, as demonstrated in a previous study.^7^

### Statistical analysis

Statistical analyses were performed using SPSS version 29 (IBM SPSS Statistics, Armonk, NY, USA). *P*-values <.05 were considered statistically significant. Comparing groups, for continuous variables, the Mann-Whitney *U*-test, and for categorical variables, the chi-square test was used. For continuous variables, means with standard deviations (SDs) are reported if normally distributed, and the medians are reported if the distribution is skewed.

The AAD and AAL growth patterns among ATAAD patients were analysed using R software (version 4.0.4). Linear, exponential, and logarithmic models were compared. The linear function was in the form f(x)=ax+b, the exponential function was f(x)=c+d×e^*x*^, and the logarithmic function was f(x)=g+h×log⁡(x). The unknown parameters *a, b, c, d, g*, and *h* were found by adjusting the curve to best fit the data. For this comparison, a linear mixed-effects model with random intercept and slope parameters was used, and a comparison between models was performed using Akaike information criteria (AIC). The model with the lowest AIC value was considered the most accurate for describing the growth pattern. An exploration of the growth patterns was performed to find the best model to estimate the ascending aortic dimensions on the day of ATAAD onset and their growth rates before ATAAD.

The AAD and AAL growth rates (mm/year) were calculated using a linear model and were used in the ATAAD risk analysis. For ATAAD patients, a linear model was also used to calculate the AAD and AAL on the day of ATAAD onset. These values were used to explore how many ATAAD patients failed to exceed preventive surgical thresholds on the day of ATAAD onset.

An error analysis was performed to estimate whether the error between measured and calculated AADs and AALs would increase while approaching the day of the ATAAD onset to ensure that the estimated ascending aortic dimensions were reliable enough to explore the number of ATAAD patients who did not exceed the preventive surgical thresholds on the day of ATAAD onset. The error analysis included ATAAD patients who had undergone at least three imaging procedures. The AAD and AAL estimations were calculated at the time of the latest image based on all measurements except the last one, using the linear model. The errors between the estimated and measured values were calculated. The time intervals between the latest image and the day of the ATAAD onset were also calculated. The correlation between the time intervals and the errors was calculated using Spearman’s rho correlation. Values > 0.50 were considered high, between 0.30 and 0.50 as moderate, between 0.10 and 0.30 as low, and smaller than 0.10 as negligible correlations.^16^

The Cox proportional hazards model was used to examine the associations between the parameters and the risk of ATAAD and to derive an equation for estimating the five-year risk of ATAAD. The assumptions of the Cox model were evaluated and found to be satisfied. Model goodness-of-fit was assessed using the Hosmer-Lemeshow test, and calibration was evaluated using the Brier score and the calibration slope. The predictive performance of the model was tested using a five-year time-dependent area under the curve (AUC) coefficient with 95% confidence intervals (CIs). AUC values of less than 0.5 were considered, as the model performed worse than random. Values between 0.5 and 0.7 were considered poor, between 0.7 and 0.8 were considered acceptable, and between 0.8 and 0.9 were considered excellent discrimination.^17^

## Results

### Baseline characteristics

**Table 1** summarizes the baseline characteristics. Patients were divided into two groups based on whether they did (ATAAD group) or did not experience ATAAD (non-ATAAD group) during the median surveillance time of 5.7 years (ranging from 1 to 19 years). The majority of the patients were male, and the mean age was 58.2 ± 12.4 years. Non-ATAAD patients had a higher weight and body mass index than ATAAD patients. Dyslipidaemia and coronary artery disease were more common among non-ATAAD patients, while ATAAD patients had other aortic diseases more commonly. ATAAD patients were more frequently smokers. ATAAD patients had the maximum AAD more frequently in the tubular aorta and non-ATAAD patients in the sinus of Valsalva.

**Table 1.** Baseline Characteristics and the Information on the AAD, the AAL, and the Growth Rates

	All (*n* = 116)	Non-ATAAD (*n* = 86)	ATAAD (*n* = 30)	*P*-value
Age (years)	58.2 ± 12.4	57.8 ± 12.3	59.4 ± 13.0	.354
Male	88 (75.9%)	68 (79.1%)	20 (66.7%)	.172
Height (cm)	176.3 ± 9.6	177.1 ± 9.3	174.2 ± 10.4	.291
Weight (kg)	86.8 ± 18.1	89.1 ± 17.1	80.3 ± 19.6	**.028**
BMI (kg/m^2^)	27.8 ± 4.6	28.3 ± 4.2	26.3 ± 5.2	.072
Hypertension	89 (76.7%)	68 (79.1%)	21 (70.0%)	.311
Diabetes	15 (12.9%)	13 (15.1%)	2 (6.7%)	.348
Dyslipidaemia	58 (50.0%)	48 (55.8%)	10 (33.3%)	**.034**
CAD	17 (14.7%)	16 (18.6%)	1 (3.3%)	**.041**
Smoking	31 (26.7%)	16 (18.6%)	15 (50.0%)	**< .001**
Family history of TAA	10 (8.6%)	6 (7.0%)	4 (13.3%)	1.000
Other aortic disease	13 (11.2%)	2 (2.3%)	11 (36.7%)	**< .001**
Genetic predisposition	4 (3.4%)	2 (2.3%)	2 (6.7%)	.279
Bicuspid aortic valve	15 (12.9%)	15 (17.4%)	0 (0.0%)	**.010**
Location of dilatation				**.003**
Sinus of Valsalva	72 (62.1%)	60 (69.8%)	12 (40%)	
Sinotubular junction	2 (1.7%)	0 (0.0%)	2 (6.7%)	
Tubular aorta	41 (35.3%)	26 (30.2%)	15 (50.0%)	
Proximal to truncus	1 (0.9%)	0 (0.0%)	1 (3.3%)	
Baseline AAD (mm)	46.5 ± 3.5	46.6 ± 3.0	46.4 ± 4.8	.794
Growth tendency of AAD	97 (83.6%)	69 (80.2%)	28 (93.3%)	.150
Growth rate of AAD (mm/year)	0.31 (0.12, 0.54)	0.24 (0.11, 0.40)	0.90 (0.37, 1.52)	**< .001**
Baseline AAL (mm)	98.2 ± 10.7	96.1 ± 0.9	104.2 ± 13.7	**< .001**
Growth tendency of AAL	99 (85.3%)	74 (86.0%)	25 (83.3%)	.767
Growth rate of AAL (mm/year)	0.78 (0.42, 1.47)	0.66 (0.37, 1.20)	1.54 (0.72, 3.00)	**< .001**

Statistically significant *P*-values (<.05) are shown in bold.

Abbreviations: ATAAD, acute type A aortic dissection; BMI, body mass index; CAD, coronary artery disease; TAA, thoracic aortic aneurysm.

### Growth patterns

**Figure 1.**
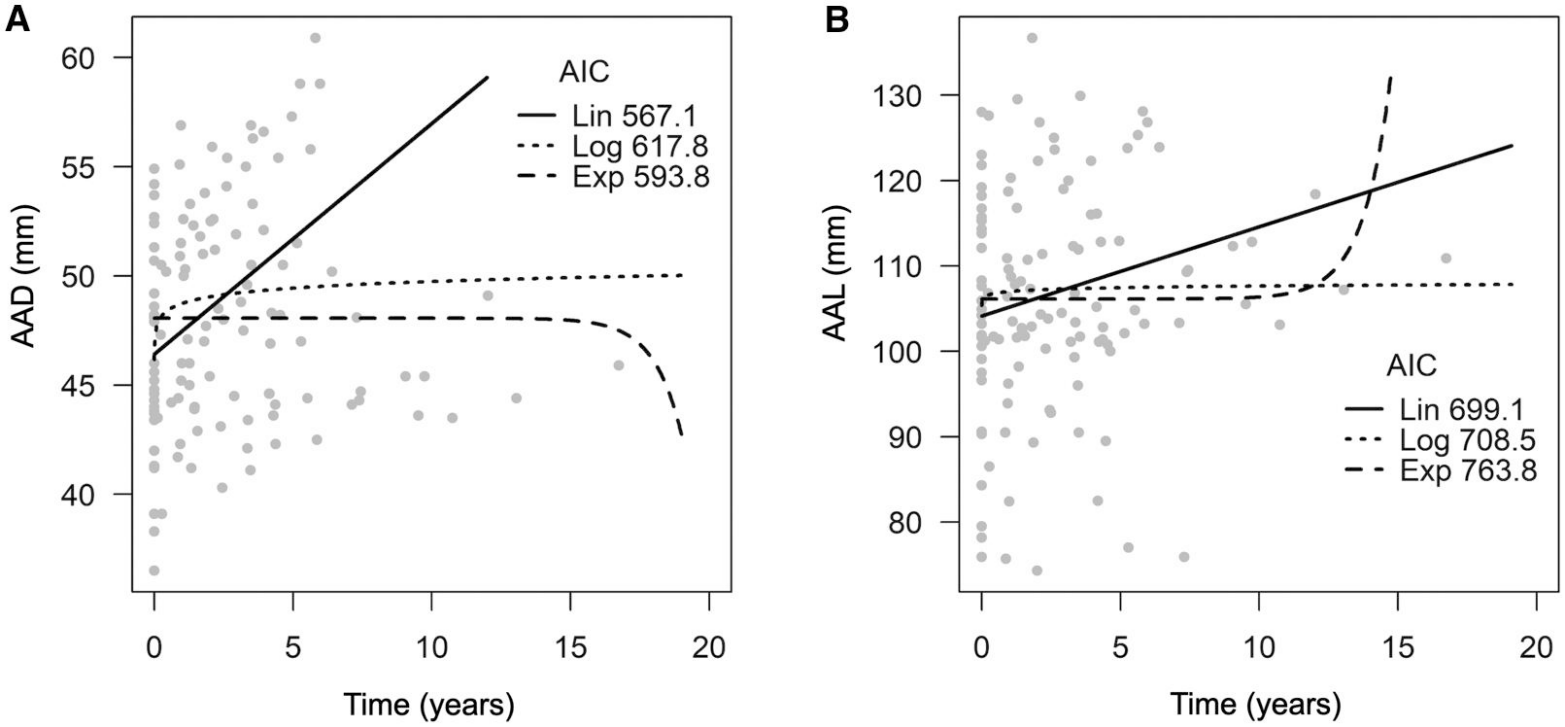
Growth patterns of the AAD (A) and AAL (B). Grey dots represent the AAD and AAL measurements of all ATAAD patients who showed growth tendencies. The linear model best describes the AAD and AAL growth patterns. Abbreviations: AAD, ascending aortic diameter; AAL, ascending aortic length; AIC, Akaike information criteria; ATAAD, acute type A aortic dissection; Exp, exponential; Lin, linear; Log, logarithmic.

The analysis of AAD and AAL growth patterns included all ATAAD patients (*n* = 30). The linear model best described the AAD and AAL growth patterns in ATAAD patients with the lowest AIC values (**Figure 1**). The logarithmic model was the most inaccurate in describing AAD growth, and the exponential model was the most inaccurate in describing AAL growth.

### Growth rates

**Figure 2.**
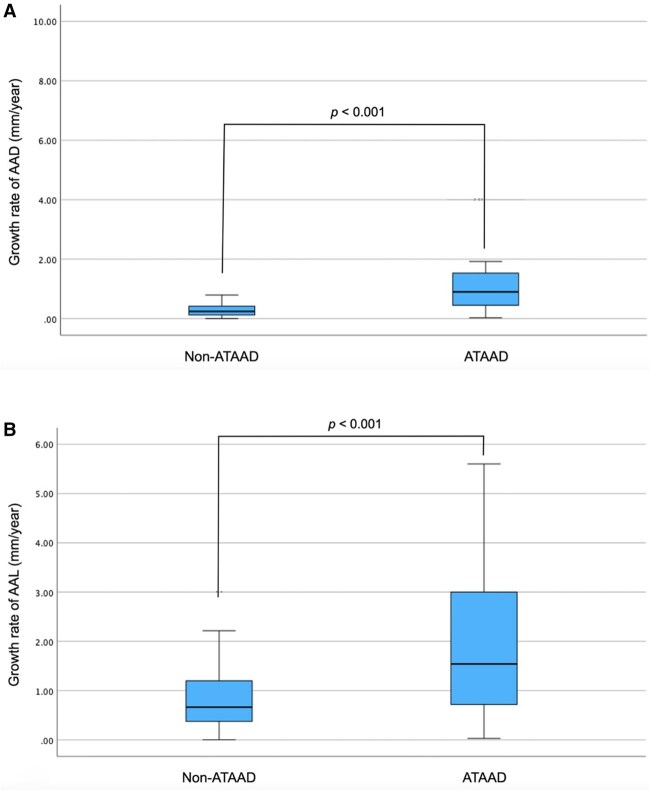
Growth rates of AAD (A) and AAL (B) among patients who showed a growth tendency during follow-up. Abbreviations: AAD, ascending aortic diameter; AAL, ascending aortic length; ATAAD, acute type A aortic dissection.

The mean initial AAD was 46.5 ± 3.5 mm. A total of 97 (83.6%) patients showed AAD growth tendencies, with a median growth rate of 0.31 mm/year. ATAAD patients showed higher AAD growth rates (0.90 mm/year vs 0.24 mm/year, *P *< .001) (**Table 1** and **Figure 2**).

The mean initial AAL was 98.2 ± 10.7 mm. ATAAD patients had longer initial AALs (104.2 ± 13.7 mm vs 96.1 ± 0.9 mm*, P *< .001). A total of 99 (85.3%) patients showed AAL growth tendencies, with a median growth rate of 0.78 mm/year. ATAAD patients showed higher AAL growth rates (1.54 mm/year vs 0.66 mm/year, *P *< .001) (**Table 1** and **Figure 2**).

### Aortic dimensions and growth rates in relation to current thresholds

**Figure 3.**
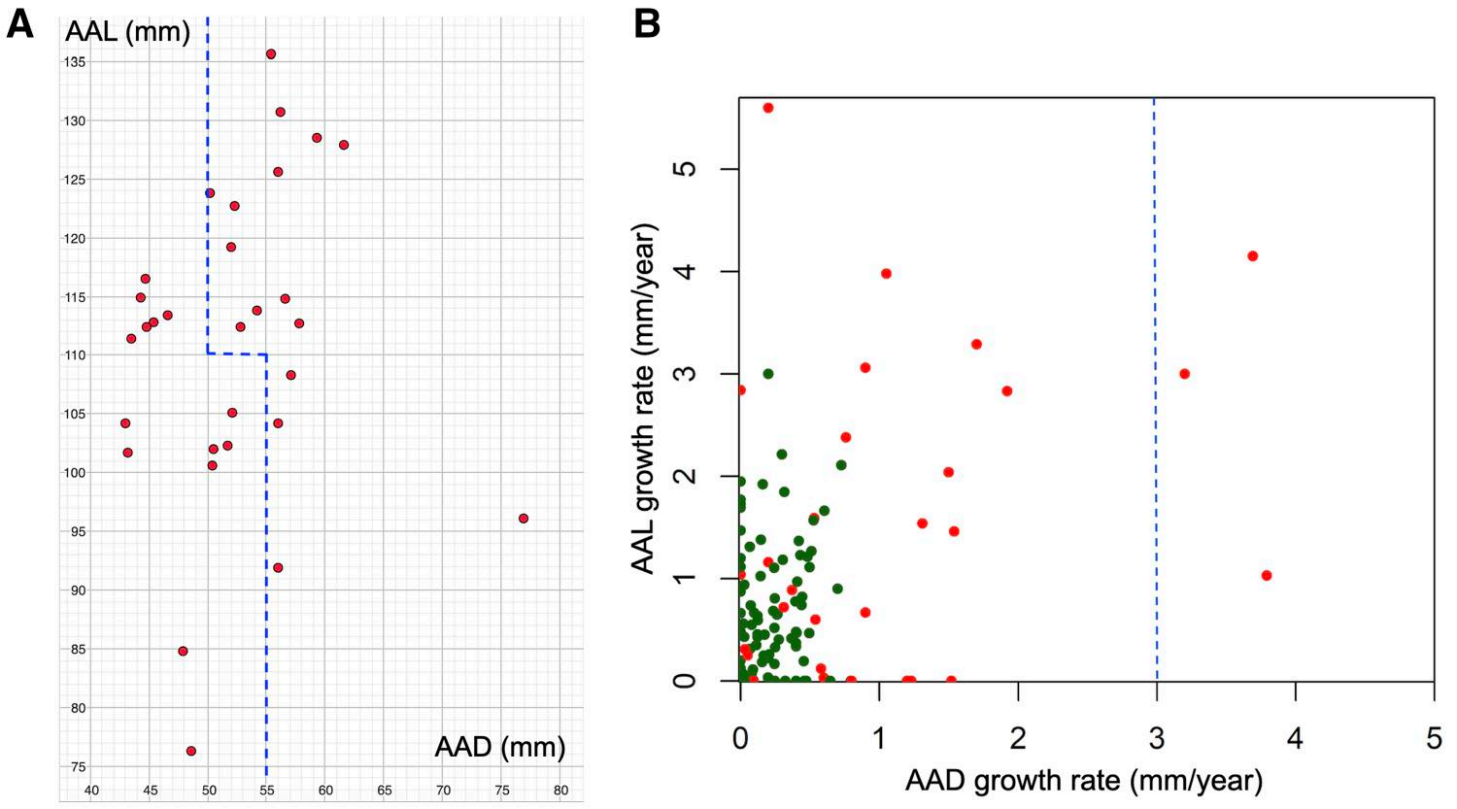
AAD, AAL, and their growth rates in relation to current thresholds. Almost half of the patients experienced ATAAD before the current thresholds (A), and only a few ATAAD patients exceeded the AAD growth rate of 3 mm/year cut-off (B). Red dots represent ATAAD patients and green dots represent non-ATAAD patients. The blue dashed lines represent the current thresholds based on the AAD, AAL, and AAD growth rate. Abbreviations: AAD, ascending aortic diameter; AAL ascending aortic length; ATAAD, acute type A aortic dissection.

Nineteen patients (63.3%) experienced ATAAD with AADs lower than 55 mm, and 12 patients (40.0%) had AALs shorter than 110 mm. Fourteen patients (46.7%) experienced ATAAD before exceeding the following thresholds regarding the AAD and AAL: the AAD > 55 mm regardless of the AAL or the AAD > 50 mm and the AAL > 110 mm (**Figure 3A**). Only 3 ATAAD patients (10.0%) and none of the non-ATAAD patients showed a growth rate higher than 3 mm/year (**Figure 3B**). Therefore, among the whole patient population, an AAD growth rate higher than 3 mm/year was rare (2.6%).

### Error analysis of estimated dimensions

**Figure 4.**
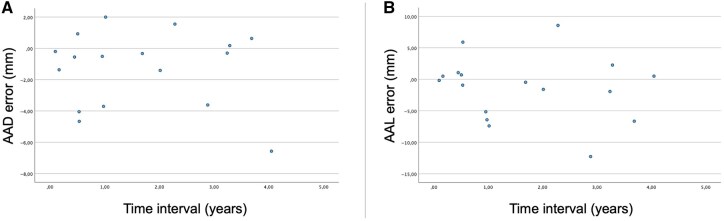
Error analysis of the AAD (A) and AAL (B). The blue dots represent the errors between the calculated and measured AADs and AALs. The AAD and AAL can be estimated as reliable enough using the linear model because the errors did not increase while approaching the ATAAD onset. Abbreviations: AAD, ascending aortic diameter; AAL, ascending aortic length; ATAAD, acute type A aortic dissection.

The error analysis included 17 ATAAD patients. The error between the estimated and measured AAD values ranged from –6.57 mm to 2.00 mm (mean error –1.29 mm). The error between the estimated and measured AAL values ranged from –12.26 mm to 8.56 mm (mean error –1.38 mm). There was no statistically significant correlation in the time intervals among the latest imaging and ATAAD onset and the AAD (correlation coefficient 0.051 and *P*-value .844) or AAL errors (correlation coefficient –0.208 and *P*-value .422); therefore, the errors of the AAD or AAL did not increase significantly when approaching the ATAAD onset (**Figure 4**). Thus, the AADs and AALs of the ATAAD patients were estimated on the day of ATAAD onset using a linear model.

### Risk for ATAAD

Because the median follow-up time was 5.7 years, the risk of ATAAD was explored for the next five years. Based on the data of this study, if the AAD and AAL and their growth rates are taken into account the individual five-year risk of ATAAD can be estimated using the risk function:


probability=1-e(-0.00032×e(lp)),

where linear predictor (lp)= 0.031×AAL+0.094×AAD+0.520×AALGR+0.006×AADGR.

The acronyms are as follows:

*AAL*: ascending aortic length (mm)

*AAD*: ascending aortic diameter (mm)

*AALGR* and *AADGR*: growth rates (mm/year) of the AAL and AAD.

**Figure 5.**
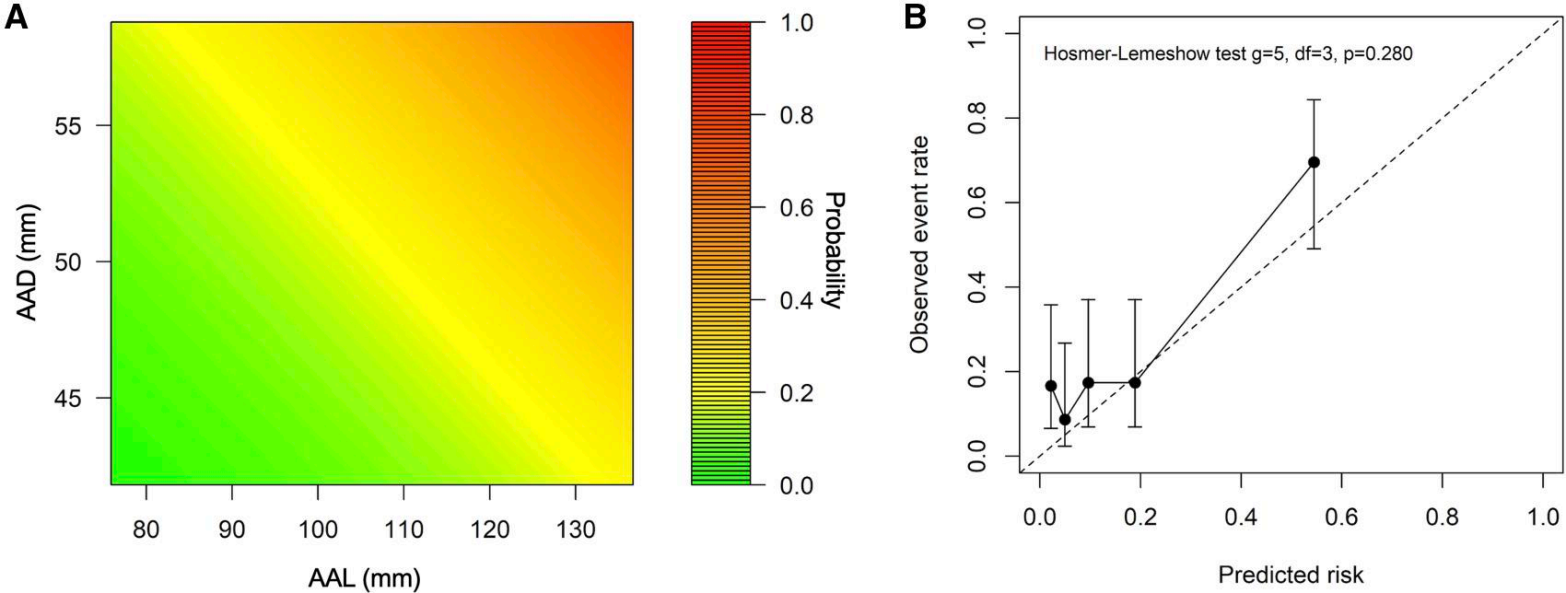
The risk of ATAAD was predicted based on the AAD and AAL and their growth rates. The risk of ATAAD increases with increasing dimensions during the one-year surveillance (A). The risk function demonstrated a good overall fit, with four out of five observed risks within the confidence intervals of the predicted risk points (B). Abbreviations: AAD, ascending aortic diameter; AAL ascending aortic length; ATAAD, acute type A aortic dissection.

**Figure 5A** illustrates how the risk of ATAAD changes with changing dimensions.

Based on this function, the factor that has the greatest impact on the ATAAD risk is the AAL growth rate (*P *< .0001), with a coefficient more than five times greater than that of the AAD, which has the second-greatest impact on the five-year ATAAD risk. Conversely, the AAD growth rate has the lowest impact on the risk of ATAAD. In univariate analysis, all four parameters were statistically significant, indicating that each variable is significant in isolation. However, in the multivariable analysis, only the AAL growth rate was statistically significant, indicating that when all variables are used in the risk analysis, the AAL growth rate affects the ATAAD risk the most (**Table 2**).

**Table 2.** Baseline Uni- and Multivariate Analysis of the Cox Proportional Hazards Model

	Univariate	*P*-value	Multivariate	*P*-value
AAL growth rate	1.64 (1.31-2.04)	**<.001**	1.68 (1.28-2.22)	**<.001**
AAL	1.05 (1.02-1.09)	**.005**	1.03 (0.99-1.07)	.112
AAD growth rate	1.16 (1.01-1.35)	**.042**	1.01 (0.82-1.24)	.954
AAD	1.17 (1.06-1.28)	**.001**	1.10 (0.98-1.23)	.111

Univariate and multivariate numbers express hazard ratios and 95% confidence intervals. Statistically significant *P*-values (<.05) are shown in bold.

Abbreviations: AAD, ascending aortic diameter; AAL, ascending aortic length.

To evaluate the model’s performance, the predicted probability of ATAAD during the follow-up was calculated. The overall model fit was good. The Hosmer-Lemeshow test supported adequate agreement between the predicted and observed probabilities, *P *= .280 (**Figure 5B**). The Brier score was 0.15, indicating that the predicted risks were close to the observed events. The calibration slope was 0.64 (95% CI [0.38-1.00]), suggesting a slight underestimation of risk at higher predicted values. The model demonstrated excellent discrimination, with a five-year time-dependent AUC of 0.83 (95% CI [0.54-0.98]).

The model with the patient’s height-indexed AAD and AAL was also explored, but indexed AAD and AAL values did not increase the discrimination, with a five-year time-dependent AUC of 0.82 (95% CI [0.50-1.00]).

## Discussion

ATAAD patients are hard to differentiate from other ATAA patients before dissection onset. In the current guidelines, the factor that has a great impact on the time for a preventive operation is the AAD, taking into account the AAD growth rate and the AAL. The cut-offs are determined as an AAD between 50 and 55 mm, an AAL >110 mm, and an AAD growth rate >3 mm/year.^2^^,^^3^ This study was created to take a closer look at evaluating the risk of ATAAD based on the AAD, the AAL, and their growth rates.

### Growth patterns

Hirose et al^18^ conducted the first study exploring the AAD growth pattern among ATAA patients and indicated the growth to be exponential. Controversially, our previous study demonstrated that the linear model best describes AAD growth among ATAA patients.^15^ Among ATAAD patients, there is a dearth of data on AAD and AAL growth patterns. In our data, the linear model best describes both growth patterns, which concurs with our previous findings.^15^ Therefore, the linear model could be used to calculate ATAAD patients’ AAD and AAL growth rates before dissection onset and to estimate the aortic dimensions at different time points.

### Growth rates

Non-ATAAD patients showed extremely low AAD growth rates (mean 0.27 ± 0.19 mm/year), as in previous studies (mean ranging from 0.2 to 2.8 mm/year).^4^^,^^5^ Among non-ATAAD patients, the mean AAL growth rate was slightly higher (mean 0.82 ± 0.62 mm/year) than in one previous study (mean 0.38 mm/year).^19^ However, ATAAD patients had significantly higher AAD growth rates before ATAAD onset, although growth rates higher than 3 mm/year were extremely rare. This concurs with previous studies.^1^^,^^4^^,^^5^ ATAAD patients also had higher AAL growth rates, which concurs with the fact that ATAAD patients have longer ascending aortas.^6^^,^^7^ Still, very little is known about the AAD and AAL growth rates among ATAAD patients before the ATAAD onset.

### Ascending aortic dimensions

ATAAD is a rare and extremely difficult-to-predict event, making it challenging to obtain imaging just before the ATAAD onset. In a few studies, ascending aortic dimensions have been estimated at the time of dissection based on pre- and post-dissection dimensions.^8^^,^^9^ This kind of study design is limited by the fact that the time between pre- and post-dissection images may be long, leading to an error in the assessment.

This study relied exclusively on pre-dissection images to estimate the AAD and AAL at the time of ATAAD onset and to assess their growth rates prior to ATAAD using a linear model. This approach reduces the impact of measurement errors. Based on the error analysis, the AAD and AAL can be reliably estimated using the linear model, even when approaching the time of the ATAAD onset.

Most of the patients (63.3%) did not exceed the thresholds of AAD for preventive surgery before the ATAAD onset, which concurs with previous studies.^8–10^ Two-fifths (40.0%) of the ATAAD patients in our study had an AAL shorter than 110 mm, which is slightly less than in one previous study.^11^ Based on the AAD and the AAL, almost half of the patients (46.7%) did not exceed the current recommendations for preventive surgery on the day of ATAAD onset. Therefore, investigating ascending aortic dimensions at the time of ATAAD onset in a larger patient cohort may help explore whether current surgical thresholds require further optimization.

### ATAAD risk evaluation

When the AAD, AAL, and their growth rates were taken into account, the five-year risk of ATAAD could be estimated with excellent confidence (AUC value 0.83). Current guidelines do not take into account the AAL growth rate when recommending preventive surgery.^2^^,^^3^ Based on our data, the AAL growth rate has the greatest impact on the risk of ATAAD compared to the other three variables, indicating that the AAL growth rate might be worth taking into account when considering preventive surgery. At the same time, the AAD, the AAL, and the AAD growth rate have a markedly lower impact on the risk of ATAAD, which concurs with the fact that most ATAAD patients fail to exceed the current preventive surgery thresholds.^8–11^ Although it has been shown that indexing the AAD and AAL to the patient’s height well predicts the risk for ATAAD,^12^^,^^13^ we were not able to show an increase in the discrimination of the risk function when using the patient’s height-indexed AAD and AAL values together with the AAD and AAL growth rates (AUC values 0.83, 95% CI [0.54-0.98] vs 0.82, 95% CI [0.50-1.00]).

The risk function we introduced was statistically created and could be used to evaluate the individual risk of ATAAD. Even though, based on our analysis, the function estimates the risk of ATAAD with good reliability, because the number of ATAAD patients was quite small, it is worth performing external validation for the risk function with a larger number of ATAAD patients. In the future, the implementation of the risk function in clinical practice might require, for example, an easy-to-use web calculator.

### Limitations

Although this study was a collaboration between two centres, the number of ATAAD patients was quite small because it turned out to be extremely rare for ATAAD patients to have any images before ATAAD onset. Due to the small number of ATAAD patients, the risk function is exploratory rather than definitive.

Because all available chest CT and MRI scans were collected regardless of the imaging indication, standardized methods for the imaging protocol could not be applied. Part of the imaging was performed without ECG gating, which might have caused artefacts, especially in the aortic root.^20^ Also, part of the imaging was performed without a contrast agent. The slice thickness of the images varied from 0.6 to 5.0 mm. These factors may have influenced the accuracy of the measurements.^21^

There was no control group in this study, so the AAD and AAL and their growth could not be compared to those of healthy people.

Inter- and intraobserver variability was not investigated. However, these have previously been shown to be low in both AAD and AAL.^7^^,^^22^

## Conclusion

Almost half of the ATAAD patients did not meet the preventive surgery thresholds based on the AAD and the AAL. An AAD growth rate higher than 3 mm/year was extremely rare. The individual five-year risk for ATAAD could be estimated with excellent confidence when using the AAD and AAL and their growth rates.

## Data Availability

The anonymized data underlying this article will be shared on reasonable request to the corresponding author.
